# Biodistribution of allogenic umbilical cord-derived mesenchymal stromal cells after fetal repair of myelomeningocele in an ovine model

**DOI:** 10.1186/s13287-022-02991-0

**Published:** 2022-07-15

**Authors:** Yoann Athiel, Justine Nasone, Lousineh Arakelian, Lionel Faivre, Anaïs Dugas, Jean-Marie Jouannic, Jérôme Larghero, Lucie Guilbaud

**Affiliations:** 1 Stem Cell Biotechnologies, U976 et Centre d’Investigation Clinique en Biothérapies CIC-BT CBT501, INSERM, Paris, France; 2 Department of Fetal Medicine, APHP, Trousseau Hospital, DMU ORIGYNE, Sorbonne University, Paris, France; 3 Unité de Thérapie Cellulaire et Centre MEARY de Thérapie Cellulaire et Génique, Saint Louis Hospital, Université Paris Cité, Paris, France

**Keywords:** Mesenchymal stromal cells, Myelomeningocele, Fetal surgery, Biodistribution, Tracking, Ovine model

## Abstract

**Background:**

Myelomeningocele (MMC) is a spinal cord congenital defect that leads to paraplegia, sphincter disorders and potential neurocognitive disabilities. Prenatal surgery of MMC provides a significant benefit compared to surgery at birth. Mesenchymal stromal cell (MSC) therapy as an adjuvant treatment for prenatal surgery showed promising results in animal experiments which could be considered for clinical use in human fetuses. Despite numerous reassuring studies on the safety of MSCs administration in humans, no study focused on MSCs biodistribution after a local MSCs graft on the fetal spinal cord.

**Aim:**

The purpose of our study was to assess the biodistribution of umbilical cord-derived mesenchymal stromal cells (UC-MSCs) at birth in lambs who had a prenatal myelomeningocele repair using a fibrin patch seeded with allogenic UC-MSCs.

**Methods:**

After isolation, UC-MSCs were tagged using a green fluorescent protein (GFP)-containing lentiviral vector. MMC defects were surgically created at 75 days of gestation and repaired 15 days later using UC-MSCs patch. Lambs were delivered at 142 days and sacrificed. DNA extraction was performed among biopsies of the different organs and q-PCR analysis was used to detect the expression of GFP (GFP DNA coding sequence).

**Results:**

In our 6 surviving lambs grafted with UC-MSCs, GFP lentivirus genomic DNA was not detected in the organs.

**Conclusion:**

These reassuring data will support translational application in humans, especially since the first human clinical trial using mesenchymal stromal cells for in-utero treatment of MMC started recently in U.S.A.

## Introduction

Myelomeningocele (MMC) is a spinal cord congenital defect which leads to paraplegia, sphincter disorders and cognitive disabilities. Prenatal repair surgery of MMC improves motor function and neurological outcomes compared to postnatal repair [1, 2]. However, this benefit remains limited since 71% of children are not able to walk independently at an average age of 7.8 years [3, 4]. Several international studies investigated the use of stem cells as an adjuvant therapy of MMC prenatal surgery [5]. Thus, very promising results using human placental-derived mesenchymal stromal cells grafted on the fetal spinal cord during surgery in the MMC ovine model have been reported [6–9]. Our group experimented the use of allogenic umbilical cord-derived mesenchymal stromal cells (UC-MSCs) in the same MMC ovine model and showed similar findings [10]. However, demonstrating the safety of MSCs graft in fetuses is essential before considering clinical application in humans. For this reason, the European Medicines Agency recommends the study of the cells kinetics, migration and persistence [11]. Despite numerous reassuring studies on the safety of MSCs administration in humans [12–14], the study of their kinetics and migration after a fetal spinal cord grafting has never been reported so far.

The objective of our study was to study the biodistribution of UC-MSCs at birth after fetal MMC surgical repair using an allogenic UC-MSCs patch in an ovine model.

## Materials and methods

### Ethics statement

This study protocol was approved by the French national committee on animal research (APAFIS#2845-2015100520053611v10) and all animals received care in strict compliance with institutional guidelines, and guidelines for the provision of standard care to laboratory animals. The study was carried out in compliance with the ARRIVE guidelines.

### Ovine MSCs production

UC-MSCs were collected from lambs delivered by cesarean section at 139 days of gestation and isolated using the explant method as previously described [10].

### GFP transduction

UC-MSCs were transduced using a green fluorescent protein (GFP)-containing lentiviral vector (DharmaconTM GIPZTM Lentiviral shRNA, Horizon Discovery, Lafayette, USA) as previously described [10]. Three days after transduction, we confirmed the presence of at least 90% GFP-tagged cells by flow cytometry analysis. Cell selection was performed using puromycin for about 10 days. GFP-tagged UC-MSCs were then cultured to obtain the required number of cells in complete medium Minimum Essential Medium alpha (MEM α) GlutaMAX™ supplement, no nucleosides (Gibco, Grand Island, USA), supplemented with 10% of Fetal Bovine Serum (FBS) (Cytiva, South Logan, USA) and 1% penicillin/streptomycin (Dutscher, Bernolsheim, France).

### Characterization of ovine UC-MSC

Characterization of UC-MSCs was previously described [10]. Briefly, it included (1) a growth analysis until passage 5 to assess the doubling time for each passage, (2) flow cytometry assay to identify the typical MSCs membrane antigen expression (CD29, CD73, CD90, CD105, CD45) and antigen expression of ovine MSCs isolated from bone marrow (CD31, CD44, CD166), and (3) analysis of multipotency to evaluate the adipogenic and osteogenic differentiations.

### Preparation of the UC-MSCs fibrin patch

Sixteen million GFP-tagged UC-MSCs were seeded into a fibrin patch containing fibrinogen (20 mg/ml) and thrombin (4 NIH units/ml) from the EVICEL® kit (Ethicon, NJ, USA) as previously described [10].

### MMC defect creation and repair in the ovine model

The MMC creation and repair were performed under general anesthesia as previously described [10]. Briefly, the MMC defect creation was performed at 75 days of gestation after laparotomy and hysterotomy. A laminectomy from L1 to L5 was performed before a removal of the dura-mater at the same level. The MMC defect was repaired at 90 days of gestation. The UC-MSCs patch was placed on the spinal cord and the skin was closed over the patch, using a Vicryl 2-0 running suture. Lambs were delivered by cesarean section at 142 days of gestational age, that is 52 days after UC-MSCs graft. Lambs’ clinical evaluation was performed at 2 h of life and the animals were sacrificed for histopathological and immunohistochemical analysis. Macroscopic examinations of brain, lungs, live, spleen and intestines were performed by cross sections of the formaldehyde fixed organ.

### Lambs tissue samples

A 1 cm by 1 cm biopsy was taken from the following organs: heart, liver, kidney, intestines, spleen, lungs, bone marrow, umbilical cord and placental cotyledons. All samples were immediately stored at − 80 °C.

### DNA extraction

Tissue samples were prepared by cryogenic grinding method using liquid nitrogen, then stored at − 20 °C. Genomic DNA was extracted using PureLink® Genomic DNA Kit (Invitrogen) according to manufacturer’s instructions with an elution volume of 100 μL. DNA was stored at − 20 °C before PCR analysis. DNA isolated from GFP-tagged UC-MSCs and non-tagged UC-MSCs served as positive and negative controls, respectively.

Quality and quantity of extracted DNA were estimated by spectrophotometry at 260 and 280 nm, respectively (A_260_/A_280_) using Nanodrop Lite® Spectrophotometer (Thermo Fischer Scientific).

### Real-time quantitative PCR analysis

RT-qPCR was performed using QuantStudio 7 Flex Real-Time PCR System (Thermo Fischer Scientific). Presence of GFP in extracted DNA was evaluated using the GFP Applied Biosystems™ TaqMan™ Gene Expression Assay (Thermo Fisher Scientific) (Assay ID: Mr03989638_mr). To ensure the accuracy of the qPCR detection, presence of an ovine reference gene, ras homolog family member B (RHOB) (Assay ID: Oa04654852_s1) was also checked. RT-qPCR was performed in triplicate in 25-µl reactions containing 2.5 μL genomic DNA (corresponding to 10 ng of genomic DNA), 12.5 μL PCR Master Mix, 8.75 μL RNase-Free Distilled Water and 1.25 μL of the Applied Biosystems™ TaqMan™ Gene Expression Assay (Thermo Fisher Scientific). DNA amplification was performed according to the manufacturer's instructions: an initial activation and denaturation step of 20 s at 95 °C followed by 45 cycles consisting of 3 s at 95 °C and 30 s at 60 °C.

### Dilution analysis

Lower limit of quantitation was determined using a dilution method. DNA coming from GFP-tagged UC-MSCs (DNA GFP+) was diluted in DNA coming from non-tagged UC-MSCs (DNA GFP-) to reproduce in vivo dilution of UC-MSCs in the different organs. We defined the threshold for detection at the dilution for which the GFP + DNA was not detected.

### DNA extraction from cellular patch

To ensure that UC-MSCs seeded in the fibrin patch kept their DNA GFP+, we performed QT-PCR analysis among DNA extracted directly from the cellular patch which contained sixteen million of GFP-tagged UC-MSCs.

## Results

### Characterization of ovine UC-MSC

Flow cytometry analysis was performed and confirmed typical MSCs antigen expression according to the International Society for Cellular Therapy definition (CD29, CD73, CD90, CD105, CD45) and antigens expression usually analyzed in the study of ovine MSCs (CD31, CD44, CD166) [15]. Analysis of MSCs multipotency was performed by confirmation of adipogenic and osteogenic differentiations of isolated cells, as previously described [10].

### Efficacy

Results of previous experimentations, demonstrating the benefit of using MSCs as adjunctive treatment for MMC fetal surgery, were already reported in the original publication [10]. The fetal loss rate was 40%, consistent with the completion of two in utero surgeries during gestation. Fetal demises usually occurred after the second fetal surgery and the condition of the aborted fetuses was not suitable for valuable tissue preservation.

### Immunohistochemical analysis

**Fig. 1:**
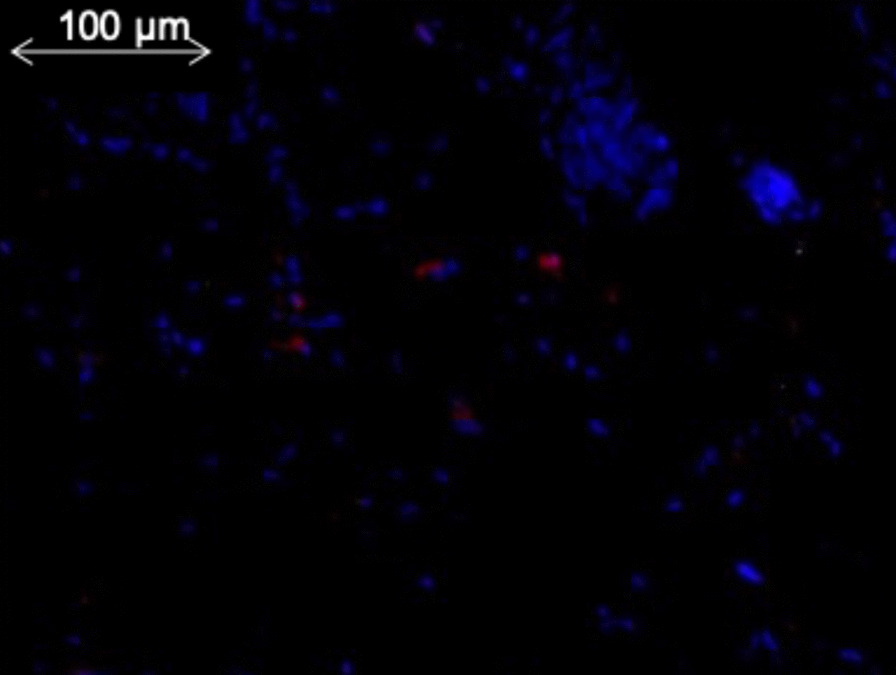
Immunohistochemical analysis of the spinal cord of a lamb who received UC-MSC patch. Few GFP-tagged were revealed in red by a primary anti-Turbo GRP antibody

We performed an immunohistochemistry analysis showing the presence of few GFP-tagged cells located in the dermis of lambs at location of the patch (Fig. 1) [10].

### Macroscopic and microscopic examinations

No tumors were observed on macroscopic and microscopic examinations of the brain, lungs, liver, spleen, and intestines of any of the six lambs [10].

### DNA extraction

DNA was purified from 2.10^6^ GFP-tagged UC-MSCs. We obtained 24,600 ng of DNA in 100 µL elution volume (246 ng/ µL). DNA was obtained from 44 samples from 6 lambs. The amount of total DNA isolated vary from 1,500 to 34,230 ng according to the sample. The DNA purity—represented by the 260/280 ratio—varies from 1.62 to 2.0.

### Validation of primers and probes

Among DNA extracted from GFP-tagged UC-MSCs, the qPCR detection was positive for GFP and RHOB probes. Among the DNA extracted from non-tagged UC-MSCs, the qPCR detection was positive for the RHOB probe but not for the GFP probe.

### Dilution analysis

Mean CT is reported in Table 1. We confirmed that the mean CT of RHOB expression was stable as the dilution proceeds but detection of GFP expression decreases gradually and finally was not detected for the dilution 1/1.10^6^. Lower limit of quantitation is 1/10^5^.

**Table 1:** Q-PCR experiment among DNA from GFP-tagged UC-MSCs diluted in DNA from non GFP-tagged UC-MSCs

Dilution		Concentration of DNA GFP + in DNA GFP-(ng/µL)	GFP applied biosystems™ TaqMan™ gene expression assay		RHOB applied biosystems™ TaqMan™ gene expression assay	
			Mean CT ± SD	CV (%)	Mean CT ± SD	CV (%)
1	1	4	26.5 ± 0.3	1.3	27.6 ± 0.07	0.2
2	1/2	2	27.9 ± 0.1	0.4	27.5 ± 0.06	0.2
3	1/5	0.8	30.8 ± 0.3	1.0	27.4 ± 0.08	0.3
4	1/10	0.4	31.3 ± 1.4	4.4	27.2 ± 0.05	0.2
5	1/100	0.04	33.9 ± 0.6	1.7	27.1 ± 0.09	0.3
6	1/1000	0.004	35.9 ± 0.8	2.2	27.2 ± 0.06	0.2
7	1/10000	0.0004	36.2 ± 0.5	1.4	27.1 ± 0.05	0.2
8	1/100000	0.00004	36.5 ± 0.9	2.5	26.9 ± 0.01	0.03
9	1/1000000	0.000004	ND	ND	27.0 + /0.04	0.1

CT, cycle threshold; SD, standard deviation; ND, not detected; GFP, green fluorescent protein, RHOB, Ras homolog gene family

### DNA extraction from cellular patch

Among DNA extracted directly from the cellular patch containing GFP-tagged UC-MSCs, detection of GFP was positive.

### Q-PCR analyses

Genomic DNA GFP + was not detected in any of the 44 samples but genomic RHOB DNA was detected in all the cases. Mean CT of QPCR analysis is reported in Table 2. All the mean CT of RHOB were in the threshold of sensibility.

**Table 2:** QPCR analysis

Organ	Lamb 1		Lamb 2		Lamb 3		Lamb 4		Lamb 5		Lamb 6	
	GFP	RHOB	GFP	RHOB	GFP	RHOB	GFP	RHOB	GFP	RHOB	GFP	RHOB
Heart	ND	28.6	ND	29.4	ND	24.0	ND	28.2	ND	27.6	NC	NC
Lung	ND	30.0	ND	28.7	ND	27.1	ND	30.2	ND	28.9	ND	28.2
Liver	ND	27.9	ND	28.1	ND	27.8	ND	28.4	ND	29.1	ND	29.2
Spleen	ND	27.8	ND	26.7	ND	31.6	ND	28.9	ND	27.5	ND	27.5
Kidney	NC	NC	ND	26.1	ND	24.3	ND	26.8	ND	31.2	ND	25.4
Bowel	ND	31.1	ND	26.9	ND	29.4	ND	26.2	ND	29.9	NC	NC
Bone marrow	NC	NC	NC	NC	NC	NC	NC	NC	ND	27.4	ND	27.0
Umbilical cord	ND	28.3	ND	27.5	NC	NC	ND	31.3	ND	31.1	ND	33.8
Placenta	ND	26.1	ND	28.2	NC	NC	ND	28.3	ND	29.7	NC	NC

NC, not collected; ND, not detected

## Discussion

In the nine organs screened in each of the six lambs grafted with allogenic UC-MSCs, no GFP lentivirus genomic DNA was detected at 52 days of the graft. These results support the safety of UC-MSCs use as an adjuvant therapy in MMC fetal surgery.

### Risk of MSCs

UC-MSCs were already used as therapeutic option in various diseases in humans [16]. Historically, stem cell therapy has been associated with an increased risk of genetic instabilities and transformation process after long-term culture [17]. However, no tumor formation has been reported in our previous experimentations and in clinical studies that focused on in vivo tumorigenesis after UC-MSCs transplantation. Recently, a large meta-analysis of 55 randomized studies with 2696 patients confirmed the safety of administrated MSCs. Especially, on longer term, there was no significant increase risk of malignancy for the MSC as compared to control groups [18]. Focusing on our way of administration, two studies reported administration of UC-MSCs directly into the spinal cord in humans after traumatic spinal cord injury. Data concerning the safety did not show any adverse event after transplantation [12, 13].

Although UC-MSCs are not very immunogenic and the fetus has an immature immunity, the allogeneic nature of the cells may explain why they do not persist in the host (immune rejection).

### MSCs in prenatal myelomeningocele therapy

Different ways of MSCs administration have been studied in prenatal myelomeningocele therapy [5]. Fauza’s group experimented intra-amniotic injections of MSCs from amniotic fluid in a retinoic acid murine model of MMC [19]. MSCs, previously labelled with a luciferase gene, were found after birth in umbilical cord, placenta, spleen and brain by luminometric analysis [20]. Although this technique is less invasive, these results could preclude application in humans.

Farmer’s team experimented human placental-derived MSCs, seeded in an extracellular scaffold, in the same surgical ovine model of MMC presented here. Through several published studies, their results suggested the benefit of MSCs in the motor function improvement [6–9]. Despite their use of MSCs transduced with GFP, no cell tracking was reported in their studies. Recently, they conducted safety evaluation of their human placental-derived MSCs in a murine model. They implanted the heterologous cells seeded on extracellular matrix into subcutaneous murine pocket. No tumor was found and MSCs did not seem to persist at the implantation site or at distance at 4 weeks and 6 months after grafting [21]. This slightly differs from our findings as we observed a survival of the MSCs 52 days after grafting [10].

Farmer’s promising results suggested the benefit of MSCs on the motor function improvement and they announced the first human clinical trial using mesenchymal stromal cells for in-utero treatment of MMC.

### Strengths and limitations

To our knowledge, this is the first biodistribution study of MSCs after local administration to the fetal spinal cord in ovine model. UC-MSCs patches were directly applied into the spinal cord in an experimental ovine model of myelomeningocele.

Several techniques exist to track UC-MSCs in transplantation experiments: luminometry, immunohistochemistry, imaging or nucleic acid amplification testing (qPCR) [20, 22, 23]. PCR analysis which is based on the amplification of the DNA is a method of choice due to its high sensitivity [24]. Our experiment of dilution shows that GFP would be detected at very low concentrations (lower limit of quantitation = 1/10^5^), below which the presence of migrating cells would probably not have negative consequences.

We recognize some limitations to our study. Lambs were sacrificed shortly after birth (2 h) which did not allow for long-term studies. Furthermore, only one biopsy of the different organ was collected so they are not analyzed in their entirety. Finally, in previous experimentations, we performed an immunohistochemistry analysis showing the presence of few GFP-tagged cells located in the dermis of lambs at location of the patch. Unfortunately, this sample was not available to perform DNA extraction and GFP screening as a positive control of the RT-qPCR analysis. This should prompt systematic dermal biopsies in further studies.

## Conclusion

This biodistribution study of grafted UC-MSCs was essential before considering a clinical application in humans, especially in the context of a fetal administration. Within the limits of our experimentations, we have shown that UC-MSCs, administered in a fibrin patch applied to the MMC defect, do not appear to disseminate to distant organs. This study is consistent with the possibility of use in humans.

## Data Availability

All data generated or analyzed during this study are included in this published article.

## Abbreviations

DNA: Deoxyribonucleic acid
GFP: Green fluorescent protein
MMC: Myelomeningocele
PCR: Polymerase chain reaction
RHOB: Ras homolog family member B
UC-MSC: Umbilical cord-derived mesenchymal stromal cell

## References

[1] Adzick NS, Thom EA, Spong CY, Brock JW, Burrows PK, Johnson MP (2011). A randomized trial of prenatal versus postnatal repair of myelomeningocele. N Engl J Med.

[2] Guilbaud L, Maurice P, Lallemant P, De Saint-Denis T, Maisonneuve E, Dhombres F (2021). Open fetal surgery for myelomeningocele repair in France. J Gynecol Obstet Hum Reprod.

[3] Houtrow AJ, Thom EA, Fletcher JM, Burrows PK, Adzick NS, Thomas NH, et al. Prenatal repair of myelomeningocele and school-age functional outcomes. Pediatrics. 2020;145(2). 10.1542/peds.2019-1544 PMC6993457 31980545

[4] Farmer DL, Thom EA, Brock JW, Burrows PK, Johnson MP, Howell LJ (2018). The management of myelomeningocele study: full cohort 30-month pediatric outcomes. Am J Obstet Gynecol.

[5] Dugas A, Larghero J, Zérah M, Jouannic JM, Guilbaud L (2020). Cell therapy for prenatal repair of myelomeningocele: a systematic review. Curr Res Transl Med.

[6] Kabagambe S, Keller B, Becker J, Goodman L, Pivetti C, Lankford L (2017). Placental mesenchymal stromal cells seeded on clinical grade extracellular matrix improve ambulation in ovine myelomeningocele. J Pediatr Surg.

[7] Wang A, Brown EG, Lankford L, Keller BA, Pivetti CD, Sitkin NA (2015). Placental mesenchymal stromal cells rescue ambulation in ovine myelomeningocele. Stem Cells Transl Med.

[8] Galganski LA, Kumar P, Vanover MA, Pivetti CD, Anderson JE, Lankford L (2020). In utero treatment of myelomeningocele with placental mesenchymal stromal cells—selection of an optimal cell line in preparation for clinical trials. J Pediatr Surg.

[9] Vanover M, Pivetti C, Lankford L, Kumar P, Galganski L, Kabagambe S (2019). High density placental mesenchymal stromal cells provide neuronal preservation and improve motor function following in utero treatment of ovine myelomeningocele. J Pediatr Surg.

[10] Guilbaud L, Dugas A, Weber M, Deflers C, Lallemand P, Lilin T, Adam C, Mebarki M, Cras A, Zérah M, Faivre L, Larghero JJJ (2021). In utero treatment of myelomeningocele with allogeneic umbilical cord mesenchymal stromal cells in an ovine model. Curr Res Transl Med.

[11] Guideline on human cell-based medicinal products, European Medicines Agency. 2008.

[12] Xiao Z, Tang F, Zhao Y, Han G, Yin N, Li X (2018). Significant Improvement of acute complete spinal cord injury patients diagnosed by a combined criteria implanted with neuroregen scaffolds and mesenchymal stem cells. Cell Transplant.

[13] Deng W-S, Ma K, Liang B, Liu X-Y, Xu H-Y, Zhang J (2020). Collagen scaffold combined with human umbilical cord-mesenchymal stem cells transplantation for acute complete spinal cord injury. Neural Regen Res.

[14] Lalu MM, McIntyre L, Pugliese C, Fergusson D, Winston BW, Marshall JC (2012). Safety of cell therapy with mesenchymal stromal cells (SafeCell): a systematic review and meta-analysis of clinical trials. PLoS ONE.

[15] Adamzyk C, Emonds T, Falkenstein J, Tolba R, Jahnen-Dechent W, Lethaus B (2013). Different culture media affect proliferation, surface epitope expression, and differentiation of ovine MSC. Stem Cells Int.

[16] Mebarki M, Abadie C, Larghero J, Cras A (2021). Human umbilical cord-derived mesenchymal stem/stromal cells: a promising candidate for the development of advanced therapy medicinal products. Stem Cell Res Ther.

[17] Barkholt L, Flory E, Jekerle V, Lucas-Samuel S, Ahnert P, Bisset L (2013). Risk of tumorigenicity in mesenchymal stromal cell-based therapies–bridging scientific observations and regulatory viewpoints. Cytotherapy.

[18] Thompson M, Mei SHJ, Wolfe D, Champagne J, Fergusson D, Stewart DJ (2020). Cell therapy with intravascular administration of mesenchymal stromal cells continues to appear safe: an updated systematic review and meta-analysis. EClinicalMedicine.

[19] Shieh HF, Tracy SA, Hong CR, Chalphin AV, Ahmed A, Rohrer L (2019). Transamniotic stem cell therapy (TRASCET) in a rabbit model of spina bifida. J Pediatr Surg.

[20] Shieh HF, Ahmed A, Rohrer L, Zurakowski D, Fauza DO (2018). Donor mesenchymal stem cell linetics after transamniotic stem cell therapy (TRASCET) for experimental spina bifida. J Pediatr Surg.

[21] Jackson JE, Pivetti C, Stokes SC, Theodorou CM, Kumar P, Paxton ZJ (2021). Placental mesenchymal stromal cells: preclinical safety evaluation for fetal myelomeningocele repair. J Surg Res.

[22] Chalphin AV, Lazow SP, Labuz DF, Tracy SA, Kycia I, Zurakowski D (2021). Transamniotic stem cell therapy for experimental congenital diaphragmatic hernia: structural, transcriptional, and cell kinetics analyses in the nitrofen model. Fetal Diagn Ther.

[23] Han L, Ma C, Peng H, Wu Z, Xu H, Wu J (2021). Define mesenchymal stem cell from its fate: Biodisposition of human mesenchymal stem cells in normal and Con-A induced liver injury mice. J Pharmacol Exp Ther..

[24] European Medicines Agency. Guideline on the quality, non-clinical and clinical aspects of gene therapy medicinal products. 2018.

